# Impact of Modern Oven Treatments on Lipid Oxidation and Vitamin E Content of Fillets from Sardine (*Sardina pilchardus*) at Different Reproductive Cycle Phases

**DOI:** 10.3390/antiox12061312

**Published:** 2023-06-20

**Authors:** Ancuta Nartea, Lama Ismaiel, Emanuela Frapiccini, Pasquale Massimiliano Falcone, Deborah Pacetti, Natale Giuseppe Frega, Paolo Lucci, Sabrina Colella

**Affiliations:** 1 Department of Agricultural, Food and Environmental Sciences, Università Politecnica delle Marche, Via Brecce Bianche, 60131 Ancona, Italy; 2 National Research Council—Institute of Marine Biological Resources and Biotechnologies (CNR-IRBIM), 60125 Ancona, Italy

**Keywords:** gonadosomatic index, mesenteric fat, steam oven, sous-vide oven, vitamin E, ω3 polyunsaturated fatty acids

## Abstract

The beneficial effects of sardine consumption can be related to the presence of bioactive compounds, such as vitamin E and ω3 polyunsaturated fatty acids. In any case, the levels of these compounds in sardine fillet depend on different factors mainly related to the diet and reproductive cycle phase of the fish as well as the technological treatments carried out to cook the fillets. The aim of the present study is two-fold: first, to evaluate changes in the total fatty acid profile, lipid oxidation, and vitamin E content of raw fillets from sardine (*Sardina pilchardus*) at different reproductive cycle phases (pre-spawning, spawning, and post-spawning); and second, to highlight how these nutritional profiles are affected by three oven treatments (conventional, steam, and sous-vide). For this purpose, raw fish was grouped into pre-spawning, spawning, and post-spawning phases according to the mesenteric fat frequency and the gonadosomatic index evaluation, and submitted to conventional (CO), steam (SO), and sous-vide (SV) baking. The ratio of EPA/DHA and vitamin E increased from post-spawning to pre-spawning, to spawning. Considering the reproductive phases, baking affected the oxidative degree differently: a CO > SO ≥ SV impact was found in the worst scenario (post-spawning), mitigated by vitamin E, to CO ≥ SO > SV in the best scenario (spawning). SV was the best treatment with high values of vitamin E in pre-spawning individuals (110.1 mg/kg). This study shows how vitamin E is correlated to the combined effect of endogenous and exogenous factors.

## 1. Introduction

Preventive nutrition is a strong tool to restrain metabolic diseases such as obesity and diabetes. A relevant and affordable food source of multiple bioactive compounds (minerals, proteins, vitamins, and essential fatty acids) is sardine (*Sardina pilchardus*, Walbaum, 1792), which is a fast-growing species, a batch spawner with a protracted spawning season and short-life cycle [1,2,3]. This species is one of the most relevant pelagic fish of the Mediterranean Sea, especially the Adriatic Sea, due to its huge ecological to higher trophic levels at the bottom-up scale as sardines are considered important components of the food web [4,5,6]; due to its economic roles [3,7], with a global volume of 3.57 million tons in 2020, which remained stable in 2021–2026 [8]; and due to its nutritional value.

Sardine fillet (edible tissue) has a high level of ω3 polyunsaturated fatty acids (ω3 PUFA), especially eicosapentaenoic (EPA) and docosahexaenoic (DHA) acids. The total content of EPA and DHA, together with the ratio of ω3/ω6 PUFA, is the most important index within the lipid quality parameters of seafood [9], showing healthy effects on the prevention of cardiovascular diseases, cancer [10,11,12,13,14], and neurodegenerative diseases [15,16,17,18,19], the potential prevention of autoimmune and rheumatoid arthritis diseases [20,21], as well as an anti-obesity effect [22]. Increasing evidence suggests the possible role of future therapy for rheumatoid arthritis based on pro-resolving lipid mediators derived from DHA (14- and 17-HDHA) and EPA (15- and 18-HEPE) such as protectins and resolvins [21]. Moreover, recent findings suggested that anti-inflammatory molecules Lipoxin A4 (LXA4), resolvins, protectins, and maresins, as well as their precursors AA/EPA/DHA, either systemically or locally, could be a potential therapeutic method to prevent and treat autoimmune diseases [20]. Furthermore, EPA and DHA have shown anti-obesity effects in mice with insulin resistance when fed with a high-fat diet enriched with 4% of these omega-3 polyunsaturated fatty acids (ω3 PUFA) [22]. Recently, the high intake of sardine ω3 PUFA and taurine was claimed to have a potential protective effect against the development of type 2 diabetes for the elderly, a subject at higher risk of this metabolic disease [23]. In addition to bioactive fatty acids, sardine fillet contains tocopherols having a techno-functional role as vitamin E activity in the human body and as a strong antioxidant in fish muscle to protect EPA and DHA, during food handling and processing [24].

In any case, the levels of bioactive compounds in sardine fillet, as well as the beneficial effects related to sardine consumption, depend on the combination of exogenous and endogenous factors. Among the exogenous factors (i.e., cooking procedures and storage condition of fillets), it is well-known that heat treatment applied to cook the fillets could accelerate the oxidation of PUFA and worsen the nutritional properties of fish fillet [25,26,27,28]. Among boiling, steaming, microwaving, grilling, pan frying, and deep frying, Zhang et al. found that only pan frying and deep frying altered the fatty acid composition of grass carp fillets, increasing the general PUFA content and decreasing the ω3/ω6 PUFA ratio due to the absorption of cooking oil [29]. Interestingly, Pacetti et al. indicated an alteration in the composition of the fish phospholipid fraction depending on the frying media, with no impact of the frying temperature on sardine samples [30]. A recent study on mild oven cooking techniques, applied to salmon (*Salmo salar*), revealed the role of inner antioxidants (coenzyme Q10, tocopherols, and astaxanthin) in food processing against fatty acid oxidation [31]. Among the endogenous factors of fish (i.e., diet, size, age, reproductive cycle, temperature, season, and geographical location), the reproduction phase of sardines causes a great variability in the fillet lipid composition. Sardines are a winter batch spawner, caught all year, with the most frequent recruitment in the autumn–winter period [12]. Sardine stores lipids from spring to autumn to allocate them to reproduction [32]. Spawning led to an increase in PUFA [33]; during the months of July to September, the contents of EPA and DHA in sardine fillets were the highest, exceeding 3.5 g per 100 g [12]. Recent studies have recognized that oxidative stress and antioxidant defences in fish are related to specific situations, including pollution, geographical area, catch season, and the reproductive phase of the fish [34,35,36,37]. Reproduction is an intensive activity that elevates the metabolic rate and likely promotes the higher formation of reactive oxygen species in fish [38,39]. As a result, antioxidant defence mechanisms (enzymatic and non-enzymatic), based also on tocopherols (especially Vitamin E), can be improved to prevent oxidative damage [40]. Moreover, environmental variability and fishing pressures can cause fluctuations in sardine reproduction [41].

In view of these considerations, in order to maximize the beneficial effects of sardine dietary consumption on human health, the variation of bioactive compound levels in sardine fillets according to the combination of both exogenous (i.e., cooking process) and endogenous (i.e., reproductive phase) factors need to be clarified.

To this purpose, the present study aimed to evaluate, for the first time, the impact of three oven treatments (conventional, steam, and sous-vide) on the lipid oxidation and vitamin E content of fillets from sardine at different reproductive cycle phase (pre-spawning, spawning, and post-spawning). Total fatty acid composition and the α-tocopherol (Vitamin E) were assessed to evaluate the fillet lipid nutritional quality, whereas the oxidative status of fillet was evaluated by monitoring the primary and secondary products of lipid oxidation.

## 2. Materials and Methods

### 2.1. Fish-Sampling Collection

Fish were sampled by commercial landings of the midwater pelagic pair trawler (“volante”) vessels in the Northern and Central Adriatic Sea (FAO Major Fishing Area 37.2.1; FAO Geographical Sub-Area [GSA] 17), located in the Central Mediterranean Sea and recognized to be an important fishing ground for several species due to its high level of productivity [42]. Sampling strategy was conducted to include specimens in the widest possible size range (from 11.5 to 14.5 cm of total length) and that were undamaged, in order to assess both the gonad maturity stages (pre-spawning, spawning, and post-spawning) as well as the presence/absence of mesenteric fat. A total of 285 specimens (123 males and 162 females) of *S. pilchardus* were collected in three fish sampling (around *n* = 100 individuals per sampling), carried out in three periods (October 2022, April 2022, and July 2022). Sex was macroscopically determined based on morphological variations of gonads related to the reproductive cycle [43].

### 2.2. Reagents and Chemicals

A standard of α-tocopherol (α-T, >95%) and solvents (acetonitrile, chloroform, *n*-hexane, methanol, *n*-propanol, and diethyl ether) at HPLC grade were purchased from Merck (Darmstadt, Germany). A standard solution containing a mixture of 37 FAME (Supelco, Bellefonte, PA, USA) was used for the identification of fatty acid methyl esters.

### 2.3. Biological Parameters, Gonadosomatic Index (GSI), and Mesenteric Fat

The total body length (LT, 0.5 cm accuracy) and total weight (W, 0.1 g accuracy) of the selected *S. pilchardus* (*n* = 285) were assessed. To identify three different reproductive cycle phases (pre-spawning, spawning, and post-spawning), the gonadal development stage was established, and gonadosomatic index (GSI) and mesenteric fat were evaluated.

At each sampling (around *n* = 100), two suitable indicators, such as the GSI and the frequency of the mesenteric fat, were estimated to evaluate the reproductive phases of the cycle of *S. pilchardus* [44,45,46]. “Pre-spawning” referred to adults whose gametes were just beginning to develop; “spawning” referred to individuals whose gametes were ready for the spawning season; and “post-spawning” referred to individuals at the end of their reproductive cycle, that are sexually mature but inactive [45,47,48,49].

The GSI provides a simple measure of the extent of gonadal development [3,33,50,51,52]. It was estimated, based on total body weight (W) and gonad weight (WG, 0.001 g accuracy), as GSI = 100 (WG/W).

The mesenteric fat is the adipose tissue surrounding the gastrointestinal tract and constitutes important lipid storage. Because of its greater ability and capability to move during the gonad maturation process, mesenteric fat plays a key role in the reproductive process of small pelagic fish. The presence of the mesenteric fat was estimated by visual assessment, to detect the frequency of the distribution of the mesenteric fat and to identify the different phases of the reproductive cycle of each individual [46,53,54,55].

### 2.4. Thermal Treatments

After the biological assessment, male and female individuals were used to constitute a unique fish fillet of 200 g—more precisely, a single layer of 15 individual fish fillets of 10–15 g, as average weight was used after the evisceration, beheading, and washing of whole sardines. For each sampling (pre-spawning; spawning, and post-spawning), raw samples were constituted as reference (200 g, in triplicate), and three oven treatments were performed: convection oven (CO), steam oven (SO), and sous-vide oven (SV). CO was performed in a preheated oven at 170 °C for 20 min. SO was achieved with the same oven by steam injection in the chamber (RH% = 100) and the sardine fillets were cooked at 70 °C for 20 min. For SV, each fillet was vacuum-packed in a polypropylene heat-resistant (up to 120 °C) bag and submitted to steam-oven cooking, with the same condition as SO. Immediately after cooking, each fillet was minced in a grinder, cooled, and subsequently used for analysis. The time and temperature applied for thermal treatments represent the real household conditions, optimized upon preliminary tests.

### 2.5. Lipid Extraction

Total lipids were isolated as reported by Orlando et al. [31]. Briefly, minced sardine samples (20 g) were added to chloroform: methanol solution (60 mL, 1:2, *v*/*v*), and homogenized. The mixture was filtered through Whatman filter paper, under a vacuum with a Buchner porcelain funnel equipped. The obtained solution was washed three times with KCl aqueous solution (20 mL, 0.88%, *w*/*v*) and the organic solvent was dehydrated over sodium sulphate and evaporated at 40 °C.

### 2.6. Vitamin E Analysis

Vitamin E analysis was performed by saponification. Sardine lipid extract (100 mg) was dissolved in a pyrogallol aqueous solution (5 mL, 6% *w*/*v*) and KOH (0.5 mL, 80% *w*/*v*), soaked, placed in a water bath (70 °C, 30 min), and, after that, cooled in ice for 10 min and added with water (3 mL). The extraction was performed three times with 5 mL of *n*-hexane, 0.5 mL, and 1 mL, agitating for 30 s and centrifuging (3600 rpm, 10 min) each time. Once the organic fractions were pooled together, they were dried with rotavapor (35 °C) and dissolved in 1 mL of n-hexane, centrifugated again if needed, and prepared for injection. Samples were loaded (2 μL, at 30 °C) on a Waters Ultra-Pressure Liquid Chromatographic Acquity system (UPLC Acquity H-Class, Waters Corporation, Milford, CT, USA) equipped with a fluorimetric detector (FLD) and an Ascentis Express Hilic (15 cm × 2.1 mm, 2.7 μm, Merck, Darmstadt, Germany) set up at 30 °C. The elution conditions and fluorimeter settings are reported elsewhere [31,56]. Vitamin E was identified by comparison of retention time with pure standards and quantified with external calibration. Vitamin E curve calibration concentration ranged from 3.25 to 80.60 mg/kg (R^2^ = 0.9979).

### 2.7. Total Fatty Acids Profile

Total fatty acid methyl esters (FAME) were obtained from sardine lipid extract through alkaline transmethylation [31]. The qualitative/quantitative analysis of FAME was performed using gas chromatography using a Varian 430-GC apparatus (Varian Inc., Palo Alto, CA, USA) equipped with a flame ionization detector (FID) and a CP-Sil88 fused silica capillary column (100 m × 0.25 mm i.d., film thickness 0.2 μm, Chrompack, Middelburg, Netherlands). Instrument and elution parameters were reported by Orlando et al. [31]. FAME was identified by comparison of retention time with the 37 FAME mixture standard solution. The fatty acid profile was expressed as a percentage of fatty acid (% FA) of the total fatty acids.

### 2.8. Primary and Secondary Products of Lipid Oxidation

Primary and secondary lipid oxidation products were quantified by determination of peroxide value (PV) and 2-thiobarbituric acid reactive substances (TBARs). According to Crowe and White [57], PV was determined and the results were expressed in meq active O_2_/kg oil [58]. TBARs assay was performed following the procedure of Mozzon and Frega [59]. The optical density of the prepared solution was measured at 532 nm by using a Varian Cary 5000 UV–Vis–NIR spectrophotometer (Agilent, Santa Clara, CA, USA). The results were expressed in μmol TBARS/g oil.

### 2.9. Statistical Analysis

All analyses were carried out in three parallel replications, and mean ± SDs were presented for the values obtained. Experimental data were subjected to one-way analysis of variance (ANOVA) to evaluate lipid oxidation and vitamin E content and their interactions during the different reproductive cycles of sardine fillets. The significance of differences was assigned at *p* < 0.05 in the Tukey test. These analyses were accomplished using Statistica software (version 14.0.1.25); MetaboAnalyst 5.0 online platform was also employed for the visualization of partial least squares-discriminant analysis (PLS-DA) of fatty acid composition and for the exhibition of box plots which are reported to show the distribution and the variation of PV and vitamin E concentrations in baked (CO, SO, and SV) fillets in pre-spawning (Pre Sp), spawning, and post-spawning (Post Sp) groups. Pearson’s correlation was performed using Excel-Microsoft Office Professional Plus 2019 (version 2304) to examine the relationship between vitamin E, PV, and TBARs in raw and baked fish fillets.

## 3. Results

### 3.1. Biological Parameters

The total body length and the total weight of the selected *S. pilchardus* (*n* = 285) averaged 13.2 cm (11.5–15.5) and 19.4 g (10.3–33.0), respectively. According to the presence of mesenteric fat and the trend of the GSI, fish samples (*n* = 285) have been grouped in three different reproductive phases, pre-spawning (*n* = 98), spawning (*n* = 100), and post-spawning (*n* = 87) (Table 1). Particularly, the pre-spawning group is characterized by the presence of mesenteric fat (100% in all individuals), and low values of the GSI (0.851 for females and 0.853 for males). Conversely, the spawning group is characterized by a low frequency of mesenteric fat (6% for females and 11% for males) and a high value of the GSI (7.061 for females and 3.927 for males). Finally, in the post-spawning group, the mesenteric fat showed an intermediate value (61% for females and 63% and females), and the GSI reached the lowest value (0.582 for females and 0.378 for males). Since similar trends of the biological indicators (frequency of the mesenteric fat and GSI) were obtained in both males and females, the subsequent analyses were performed by taking into account the fish fillets of all individuals together.

### 3.2. Assessment of the Fatty Acid Composition, Vitamin E Content, and Oxidative Status of Raw Fillets from Sardine at Different Phases of the Reproductive Cycle

The total fatty acid composition (FA), peroxide value (PV), 2-thiobarbituric acid reactive substances (TBARs), and α-tocopherol (α T, vitamin E) were investigated in raw sardines at different reproductive phases (pre-spawning, spawning, and post-spawning).

**Figure 1:**
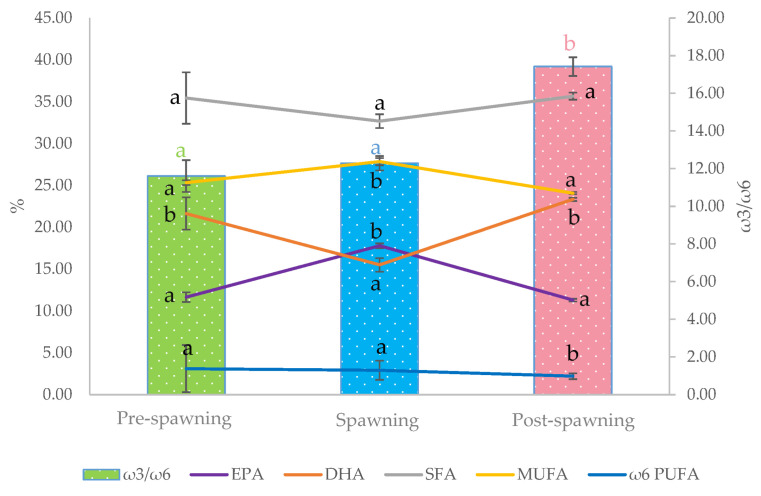
Evolution of SFA, MUFA, ω6 PUFA, EPA, and DHA contents (weight % of total fatty acids), and ω3/ω6 PUFA ratio during the reproductive cycle is reported as three phases: pre-spawning, spawning, and post-spawning. Data are expressed as mean value ± standard deviation (*n* = 3). Different letters per variable mean statistical difference (*p* < 0.01).

The results showed that the total FA composition of fillets varied with the reproductive phases, in compliance with the biological parameters of raw sardines (Appendix A, Table A1). The fatty acid composition of spawning fillet was markedly different from the other ones. Greater variations were noticed for the EPA and DHA levels (Figure 1). Although the total ω3 PUFA content did not change according to the reproductive phase, the EPA significantly increased in spawning samples, in opposition to DHA, which significantly decreased. As a result, the spawning fillet contained similar amounts of EPA and DHA, whereas in the pre- and post-spawning samples, the EPA/DHA ratio was around 0.5.

Unlike the total ω3 PUFA level, the total ω6 PUFA level showed significant differences. The post-spawning samples presented a significantly lower level of ω6 PUFA, especially of arachidonic acid (ARA, C20:4 ω6), compared to the pre-spawning and spawning samples. Consequently, in post-spawning fillets, the ω3/ω6 PUFA ratio was significantly higher than in the other samples. It increased, with an exponential trend, from the pre-spawning and spawning (11.5) to post-spawning (17.22) phase. As result, the post-spawning fillets stand out with the highest content of DHA and lowest content of arachidonic acid.

**Figure 2:**
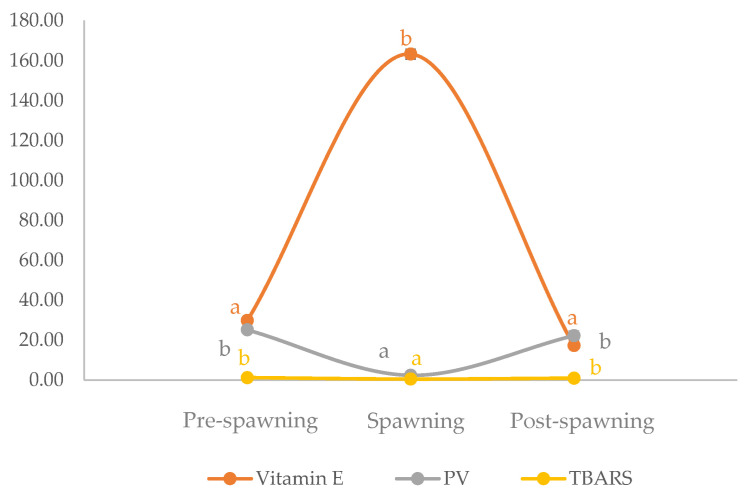
Evolution of PV, TBARS, and vitamin E during the reproductive cycle is reported as three phases: pre-spawning, spawning, and post-spawning. Data are expressed as mean value ± standard deviation. Different letters per variable means statistical difference (*p* < 0.01). PV = meqO_2_/kg oil; TBARS = µmol/g oil; vitamin E = mg/kg oil.

Considering the oxidation degree (Figure 2) of sardine fillets evaluating primary and secondary products (PV, TBARSs), the highest PV value was assessed in pre-spawning fillets (25.0 ± 1.9 meqO_2_/kg oil), followed by post-spawning (22.5 ± 2.3 meqO_2_/kg oil) and spawning ones (2.1 ± 0.8 meqO_2_/kg oil), with very low values. Simultaneously, the spawning period led to the lowest TBARs value (0.5 ± 0.1 μmol/g oil) in sardine fillets. As a result, the spawning period presented the lowest degree of oxidation.

Concerning the antioxidant content, vitamin E significantly increased from post-spawning (17.3 ± 0.6 mg/kg oil) or pre-spawning (29.8 ± 1.5 mg/kg oil) to spawning individuals (133.2 ± 20.3 mg/kg oil) (Figure 2).

### 3.3. Influence of Thermal Treatments on Fatty Acid Composition, Vitamin E Content, and Oxidative Status of Fillets from Sardine at Different Phases of Reproductive Cycle

The impact of mild oven treatments (steam and sous-vide) and conventional oven treatment on the fatty acid composition, lipid oxidation, and vitamin E content of sardine fillets were evaluated. Different technological parameters were under consideration such as temperature (170 °C vs. 70 °C), heat transfer means (hot air vs. steam), and diffusivity of oxygen (atmospheric pressure vs. under vacuum condition), at the same time of baking (20 min) per CO, SO, and SV treatments.

**Figure 3:**
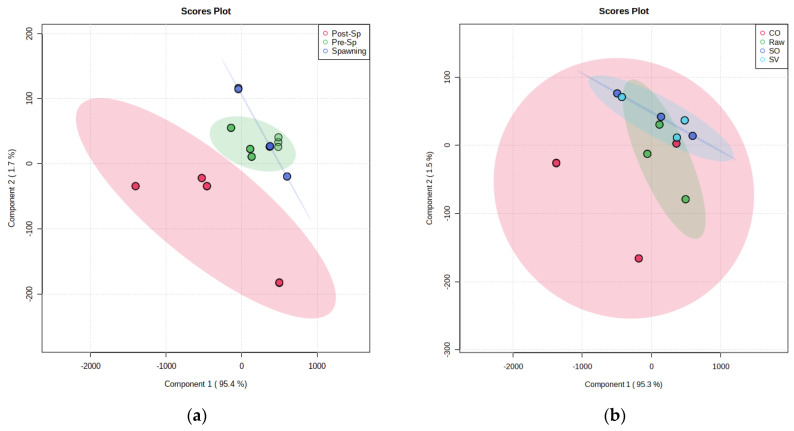
Partial least squares-discriminant analysis (PLS-DA) of fatty acid composition (weight % of total fatty acids) based on (**a**) reproductive cycle stage (pre-spawning, Pre-Sp; spawning; and post-spawning, Post-SP) and on (**b**) oven treatment (CO, SO, and SV). CO = conventional oven; SO = steam oven; SV = sous-vide oven.

Regarding the fillet FA profile, it was not affected by any treatment as few statistical differences were evidenced among raw and cooked samples, without a clear implication of the oven effect, as resulted in PLS-DA (Figure 3).

**Figure 4:**
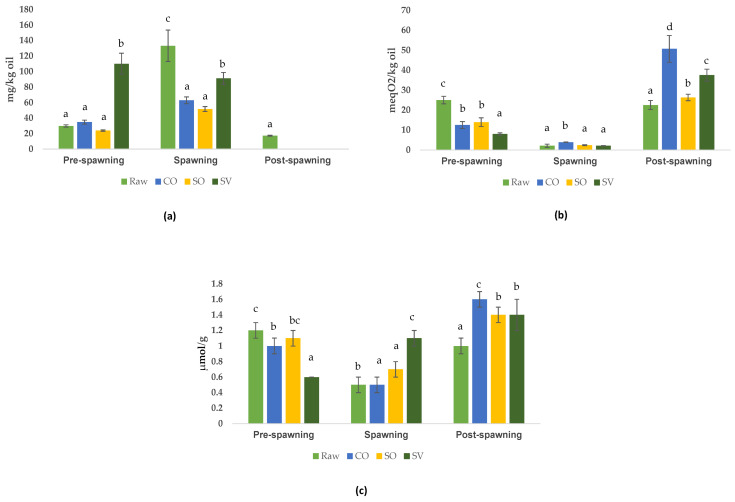
Evolution of (**a**) vitamin E (mg/kg oil), (**b**) PV (meqO_2_/kg oil), and (**c**) TBARS (µmol/g oil), in pre-spawning, spawning, and post-spawning sardines, raw and baked with different techniques: conventional oven (CO), steam oven (SO), and sous-vide (SV) oven. Values are means of three replicates ± standard deviation. Bars having different lowercase letters are significantly different at *p* < 0.05 within the same reproductive phase.

Differently, the oxidative status (Figure 4) worsened in the function of the combination of the reproductive phase and technological parameters of the oven treatment.

Considering the post-spawning samples, all thermal treatments affected the lipid oxidation and the antioxidant level in fillets, with a significant increase (*p* < 0.05) of the PV and TBARS levels. In any case, among the thermal treatments, CO led to the worst scenario of oxidation in comparison with SO and SV.

Although the raw fillet from pre-spawning sardine presented an oxidative degree (PV and TBARS levels) comparable to post-spawning raw fillet, the impact of the oven treatments resulted in markedly different outcomes. Unlike what we have seen in post-spawning, all treatments led to a significant reduction of PV and TBARs in pre-spawning fillets. The SV treatment exhibited the strongest reduction. The impactful parameter on oxidation by-product formation was the oxygen removal in SV. The temperature 170 °C (CO) vs. 70 °C (SO, SV) was not relevant in pre-spawning fillets.

The impact of the oven treatment on spawning fillets was different from that revealed for the other fillets. In fact, SV did not alter the PV value, whereas it markedly increased the TBARS. Conversely, CO did not alter the TBARS but enhanced the PV value. The SO enhanced both PV and TBARS.

Focusing our attention on the vitamin E content, all the thermal treatments (CO, SO, and SV) exhibited a loss of vitamin E in both post-spawning and spawning sardine fillets compared to the raw samples. In post-spawning samples, after the oven treatment, no vitamin E was detected anymore. On the contrary, in pre-spawning fillet, the SV treatment led to an increase in the vitamin E level. As a result, pre-spawning fillet treated with SV presented the significantly highest Vitamin E content (110.1 ± 13.6 mg/kg) in comparison with that submitted to CO (34.8 ± 2.6 mg/kg) and SO (23.8 ± 0.9 mg/kg). Similarly, SV caused the lowest loss of vitamin E in comparison to SO and CO.

**Figure 5:**
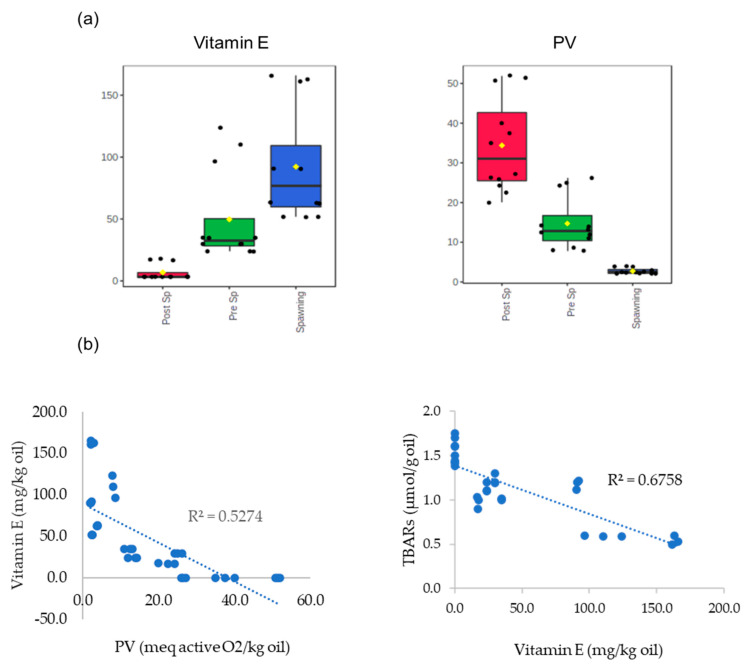
Box plots of normalized concentrations of PV and vitamin E of baked (CO, SO, and SV) fillets in prespawning (Pre Sp), spawning, and post-spawning (Post Sp) groups (**a**). CO = conventional oven; SO = steam oven; SV = sous-vide oven. Pearson’s correlation of vitamin E/PV, vitamin E/TBARs of all raw and baked samples (**b**).

In general, after baking, the influence of the reproductive phase on sardine levels of vitamin E and PV is demonstrated by the bar plots of Figure 5a, where the concentrations of the compound of raw plus cooked samples were normalized. In the raw and cooked samples, vitamin E showed an opposite correlation with PV and TBARs.

## 4. Discussion

*Sardine pilchardus* is recommended as a source of bioactive compounds (EPA, DHA, and vitamin E), but the estimation of those compounds in raw and cooked sardine fillet has to take into account the joint effect of endogenous (reproduction cycle which varies by area and season) and exogenous (thermal treatments adopted for the cooking process) factors [60,61]. Additionally, when the oven treatments are applied, the impact on the nutritional quality of cooked fillet depends on technological parameters such as the temperature, time, oxygen diffusivity, and heat transfer mean affecting the rapidity and homogeneity of the treatment, and that is in line with other studies which suggested that commonly used cooking methods may not have a similar impact on the PUFA content of various fish species (e.g., sea trout, herring, rock sole, and cod) [62]. Modern ovens are designed to allow the consumer and restaurant operators to apply traditional (conventional oven) together with mild baking treatments such as the steam oven and sous-vide oven, playing on the technological parameters.

On this matter, in the present study, the impact of different oven treatments (conventional, steam, and sous-vide) was assessed on the lipid nutritional quality and oxidative status of sardine fillet at different reproduction cycle phases. As a result, it is plausible to assume the effect of modern oven treatments on sardine fillets having different lipid compositions. It is well-known that the lipid composition is strictly related to the reproductive phase of the fish [33].

Firstly, the sardines were clustered into groups according to the reproduction cycle phase (pre-, post-, and spawning). The assessments on raw sardine followed the trend of biological indices (mesenteric fat and GSI), using precise indicators more than the season caught to identify the exact reproductive cycle phase of the fish. After that, the analysis of the biological and chemical parameters of raw sardine were performed. Our findings confirmed that during the post-spawning phase, the lipid storage in sardines, represented by the % mesenteric fat frequency, was consumed for gonad development, as revealed by the high GSI values. In the pre-spawning phase, the sardine accumulates lipids and vitamin E from the diet until the spawning moment, where the highest peak of EPA and vitamin E were found. According to Simat et al. [12], our result showed a ω3/ω6 PUFA ratio around 11.5 in pre-spawning fillets. Zorica et al. found the best condition in sardines at the beginning of the spawning season and the EPA/DHA ratio attains the value of 1 when the ovary released the mature egg batch [1].

The fish may be attempting to lower oxidative damage by consuming more antioxidants to produce eggs of higher quality [37]. This also explains our findings, since vitamin E accumulated in spawning was consumed, as low levels were found in post-spawning. The spawning phase is considered an effort for fish, causing oxidative stress, but the vitamin E was sufficient in the examined samples to limit the formation of primary (PV) and secondary oxidation products (TBARS).

A lower quality of fat (EPA and DHA), oxidative stability (PV and TBARS), and lower content of vitamin E were found in the post-spawning fillets. Similar to what was reported by Ahongo et al., significant changes in the quality of the rainbow trout flesh were noticed after 8 weeks of spawning [63].

The raw assessment was used to compare the results of oven-treated sardine fillet; however, the comparison can have some limitations because uncooked sardine has a structure different from cooked sardine. Cooking/baking causes protein denaturation, liquid and lipid leaching, solid concentration after water evaporation, the inactivation of enzymes (i.e., tocopherols oxidase), and the inactivation of microbial spoilage. The extent of these phenomena depends on the technological parameters of the cooking techniques. Therefore, the level of fatty acids and vitamin E could depend not only on heat degradation [64].

The fatty acid profile of sardine has not been significantly altered by the oven treatments. It is difficult to alter the FA profile by cooking, except for pan frying and deep frying [65]. Some observed changes were not homogeneous as some FA decreased, and some increased, as reported by García-Arias et al. [66]. In fact, this high complexity in the FA profile belongs to different factors according to the animal species and diet, and, as a consequence, food authentication based on fatty acid profile poses a great challenge; hence, they are good indicators for lipid quality [67].

The FA of the baked sardines (200 °C, 20 min), as well as the salmon (180 °C, 20 min), were well-preserved [31,65]. However, looking at the advanced oxidative status and poor antioxidant content of post-spawning sardines, in the worst scenario, the PUFA ω3 level decreased in CO and increased in SV, but this difference was not statistically evident. It is worth noting that the heat transfer of CO is less controlled and homogeneous because is less rapid than steam, leading to non-homogenous areas on the fillets [68]. Non-homogeneous oxidized parts may cause variability, limiting the statistical evidence.

As a general consideration, the negative baking impact on raw fillet’s oxidative status was as follows: CO > SO ≥ SV, found in the worst scenario (post-spawning), which can be explained by the effect of post-spawning on the quality characteristics [63] beside the positive effect of SO and SV in reducing the oxidative status in fish [31]. In the function of the reproductive phase of raw fish, this difference was mitigated by the vitamin E content to a CO ≥ SO > SV as the best scenario (spawning), considering the protective impact of vitamin E in this phase against oxidation [69]. The baking of sardine fillets at 170 °C for 20 min alters their quality. All the analyses confirmed that the worst oxidative state degree was found in traditionally baked sardines, while SO (70 °C, 20 min, atmospheric pressure) and particularly SV (70 °C, 20 min, under vacuum) showed better oxidative and antioxidant stability. These results are in correspondence with a similar study conducted on salmon fillets [31].

The role of vitamin E in counteracting oxidation during the reproductive phases of sardine is evident and expected, and of great impact also during traditional and mild oven treatments. The negative correlation of the PV/vitamin E of raw and baked sardines is strong across all baking techniques (CO, SO, and SV) and reproductive phases (pre-spawning, spawning, and post-spawning). Our finding reinforces the importance of antioxidants during cooking techniques against oxidation. An inverse correlation between the vitamin E level and oxidized CoQ_10_ and PV was found in salmon baked in a similar condition to our experiments: CO (180 °C, 20 min); SO (65 °C, 20 min); and SV (65 °C, 20 min) treatments [31]. Tocopherols in salmon were not degraded at all as with sardines. All the tested procedures registered a decrement of vitamin E, except for the pre-spawning SV sample. The authors hypothesized about the protection of carotenoids against lipid oxidation in salmon [31]. For our results, let us suppose the impact of CO can be mitigated by adding to the fish fillet preparation exogenous antioxidants such as vitamin E [24] and plant extracts (eugenol) with an antioxidant and antimicrobial effect [70], especially for sardine in the post-spawning phase. Recently, in the rainbow trout (*Oncorhynchus mykiss*), the role of tocopherols and carotenoids was explicated even in alternative and sustainable aquafeeds composed of 20% of *Hermetia illucens* prepupae. By the modulation of the rearing substrate composed of coffee silverskin and spirulina microalgae, *Hermetia illucens* accumulated carotenoids and tocopherols, used later in feeding the rainbow juveniles. It was suggested that most dietary carotenoids were used in rainbow trout to avoid inflammation in response to alternative aquafeeds, rather than accumulating them in the flesh [71].

## 5. Conclusions

Based on the results, all investigated oven treatments did not alter the bioactive fatty acid content (EPA and DHA), while they changed the oxidative status and decreased the vitamin E content in sardine tissue. However, the extent of the impact varied not only according to the different operative conditions (temperature, oxygen diffusivity, and heat transfer mean) of the treatment but also according to chemical features of the fillet related to the sardine reproductive cycle (pre-spawning, spawning, and post-spawning).

It is noteworthy that the impact of all oven treatments on the oxidative status of fillets with the highest content in vitamin E, such as spawning fillets, was weaker than that revealed in pre- and post-spawning fillets.

This study provides deeper knowledge regarding the impact of these mild treatments on sardine lipid oxidation since limited information exists in the literature. From a practical standpoint, these findings can assist the fish-farming industry in making more informed decisions about the appropriate use of sardines based on their reproductive stage, whether it is directing them to the market or for egg production, while considering the quality level of each stage. Furthermore, this study could serve as a starting point for researchers exploring the potential of a post-spawning rearing period that facilitates the restoration of desirable fillet quality.

## Figures and Tables

**Table 1:** Frequency of the mesenteric fat and GSI observed in *S. pilchardus* fillets, grouped in three reproductive phases (pre-spawning, spawning, and post-spawning).

	Pre-Spawning		Spawning		Post-Spawning	
Individuals	Frequency of Mesenteric Fat (%)	GSI	Frequency of Mesenteric Fat (%)	GSI	Frequency of Mesenteric Fat (%)	GSI
Females	100 (*n* = 53)	0.851	6 (*n* = 63)	7.061	61 (*n* = 46)	0.582
Males	100 (*n* = 45)	0.853	11 (*n* = 37)	3.927	63 (*n* = 41)	0.378
Total	100 (*n* = 98)	0.852	8 (*n* = 100)	5.905	62 (*n* = 87)	0.486

## Data Availability

The data presented in this study are available in the article.

## Appendix A

**Table A1:** Fatty acid composition (weight % of total fatty acids) of raw (pre-spawning, spawning, and post-spawning) and baked (CO, SO, and SV) sardine fillets. CO = conventional oven; SO = steam oven; SV = sous-vide oven.

	Pre-Spawning				Spawning				Post-Spawning			
Fatty Acids (%)	Raw	CO	SO	SV	Raw	CO	SO	SV	Raw	CO	SO	SV
C14:0	8.8 ± 1.3	7.8 ± 0.1	6.9 ± 0.1	8.2 ± 0.2	10.2 ± 0.3	10.2 ± 0.1	9.2 ± 0.0	10.3 ± 0.3	8.0 ± 0.3	7.0 ± 1.0	7.0 ± 0.5	7.0 ± 0.1
C16:0	22.4 ± 1.7	22.1 ± 0.2	22.5 ± 0.1	21.3 ± 0.2	18.3 ± 0.5	18.2 ± 0.1	18.7 ± 0.1	18.6 ± 0.2	22.3 ± 0.2	22.9 ± 2.9	22.4 ± 1.1	21.6 ± 0.1
C18:0	4.3 ± 0.1	4.0 ± 0.0	4.3 ± 0.1	4.2 ± 0.0	4.2 ± 0.0	4.3 ± 0.0	4.2 ± 0.0	4.2 ± 0.0	5.4 ± 0.1	4.8 ± 0.6	5.3 ± 0.1	5.3 ± 0.0
**ΣSFA**	**35.4 ± 3.1**	**34.0 ± 0.4**	**33.7 ± 0.1**	**33.7 ± 0.4**	**32.7 ± 0.8**	**32.7 ± 0.2**	**32.1 ± 0.1**	**33.1 ± 0.5**	**35.7 ± 0.4**	**33.7 ± 4.4**	**34.7 ± 1.6**	**34.0 ± 0.1**
C16:1 Δ9	7.8 ± 0.1	6.7 ± 0.4	6.2 ± 0.1	7.2 ± 0.4	11.3 ± 0.5	11.8 ± 0.1	10.8 ± 0.2	11.8 ± 0.1	7.1 ± 0.1	6.0 ± 0.8	6.9 ± 0.4	6.5 ± 0.1
C18:1 Δ9cis	6.8 ± 0.1	8.8 ± 0.0	8.2 ± 0.1	7.8 ± 0.0	6.4 ± 0.1	7.1 ± 0.0	5.6 ± 0.0	6.5 ± 0.0	8.6 ± 0.1	7.6 ± 0.9	9.1 ± 0.1	7.9 ± 0.0
C18:1 Δ11	3.6 ± 0.2	3.1 ± 0.0	2.9 ± 0.0	3.2 ± 0.0	5.4 ± 0.1	5.8 ± 0.0	5.7 ± 0.0	5.6 ± 0.0	3.0 ± 0.0	2.6 ± 0.3	3.0 ± 0.0	2.9 ± 0.0
isomer C20:1	2.0 ± 0.1	2.6 ± 0.0	2.6 ± 0.0	3.0 ± 0.1	1.0 ± 0.1	1.3 ± 0.0	1.2 ± 0.0	0.9 ± 0.0	0.3 ± 0.0	0.3 ± 0.0	0.3 ± 0.0	0.4 ± 0.0
C20:1 Δ11	1.9 ± 0.0	2.2 ± 0.0	2.0 ± 0.1	2.0 ± 0.0	2.3 ± 0.1	2.1 ± 0.0	2.2 ± 0.0	2.3 ± 0.0	3.9 ± 0.1	3.6 ± 0.5	3.4 ± 0.0	3.8 ± 0.0
C22:1 Δ11	2.0 ± 0.2	2.4 ± 0.0	2.6 ± 0.0	3.1 ±0.1	0.5 ± 0.1	0.7 ± 0.0	0.8 ± 0.0	0.1 ± 0.1	0.1 ± 0.0	0.1 ± 0.0	0.1 ± 0.0	0.1 ± 0.1
C22:1 Δ9	1.3 ± 0.6	1.0 ± 0.0	1.0 ± 0.0	1.0 ± 0.1	0.9 ± 0.0	0.9 ± 0.0	0.9 ± 0.0	1.0 ± 0.0	0.9 ± 0.1	0.9 ± 0.0	0.9 ± 0.0	1.0 ± 0.0
**ΣMUFA**	**25.3 ± 0.3**	**26.7 ± 0.4**	**25.5 ± 02**	**27.4 ± 0.3**	**27.8 ± 0.4**	**29.7 ± 0.1**	**27.3 ± 0.2**	**28.2 ± 0.1**	**24.1 ± 0.1**	**27.8 ± 9.0**	**23.9 ± 0.4**	**22.4 ± 0.1**
C18:2 Δ9,12ω6	1.8 ± 0.1	1.9 ± 0.0	2.0 ± 0.0	2.0 ± 0.0	1.4 ± 0.0	1.2 ± 0.1	1.3 ± 0.1	1.2 ± 0.1	1.7 ± 0.1	1.7 ± 0.2	1.6 ± 0.0	1.7 ± 0.0
C18:3 Δ9,12,15ω3 + C18:4 Δ6,9,11,15 ω3	1.0 ± 0.4	1.5 ± 0.2	1.0 ± 0.4	1.3 ± 0.2	0.8 ± 0.2	0.9 ± 0.2	1.2 ± 0.0	1.2 ± 0.0	2.0 ± 0.1	1.6 ± 0.2	1.8 ± 0.0	1.9 ± 0.0
C20:2 Δ14 ω6	0.4 ± 0.0	0.4 ± 0.1	0.4 ± 0.0	0.4 ± 0.0	0.3 ± 0.0	0.3 ± 0.0	0.3 ± 0.0	0.3 ± 0.0	0.2 ± 0.0	0.2 ± 0.0	0.2 ± 0.0	0.2 ± 0.0
C20:4 Δ5,8,11,14ω6 (ARA)	1.3 ± 0.1	1.2 ± 0.0	1.2 ± 0.0	1.2 ± 0.0	1.6 ± 0.0	1.5 ± 0.0	1.5 ± 0.0	1.5 ± 0.0	0.5 ± 0.0	0.5 ± 0.0	0.5 ± 0.0	0.6 ± 0.0
C20:5 Δ5,8,11,14,17ω3 (EPA)	11.6 ± 0.6	10.7 ± 0.1	10.1 ± 0.1	11.4 ± 0.2	17.8 ± 0.3	18.2 ± 0.2	16.7 ± 0.1	18.0 ± 0.2	11.3 ± 0.2	9.9 ± 1.2	11.2 ± 0.3	11.1 ± 0.0
C22:5 Δ7,10,13,16,19ω3	1.5 ± 0.1	1.5 ± 0.0	1.4 ± 0.0	1.6 ± 0.0	2.2 ± 0.1	2.3 ± 0.0	1.9 ± 0.0	2.2 ± 0.0	1.3 ± 0.0	1.1 ± 0.1	1.3 ± 0.1	1.3 ± 0.0
C22:6 Δ4,7,10,13,16,19ω3 (DHA)	21.6 ± 1.9	22.2 ± 0.5	24.8 ± 0.1	21.1 ± 0.5	15.5 ± 0.8	13.3 ± 0.2	17.8 ± 0.1	14.2 ± 0.2	23.3 ± 0.2	23.5 ± 3.0	24.7 ± 1.6	26.7 ± 0.1
**ΣPUFA**	**39.3 ± 2.8**	**39.3 ± 0.5**	**40.8 ± 0.3**	**38.9 ± 0.5**	**39.5 ± 1.2**	**37.6 ± 0.3**	**40.6 ± 0.1**	**38.6 ± 0.5**	**40.3 ± 0.3**	**38.5 ± 4.8**	**41.4 ± 2.1**	**43.6 ± 0.2**
**Σω3 PUFA**	**35.8 ± 2.8**	**35.8 ± 0.6**	**37.2 ± 0.3**	**35.4 ± 0.5**	**36.2 ± 1.1**	**34.7 ± 0.2**	**37.5 ± 0.1**	**35.6 ± 0.4**	**37.9 ± 0.4**	**36.1 ± 4.5**	**39.1 ± 2.1**	**41.1 ± 2.3**
**Σω6 PUFA**	**3.1 ± 0.0**	**3.1 ± 0.0**	**3.2 ± 0.0**	**3.1 ± 0.0**	**2.9 ± 0.1**	**2.6 ± 0.1**	**2.8 ± 0.1**	**2.7 ± 0.1**	**2.2 ± 0.0**	**2.2 ± 0.3**	**2.1 ± 0.0**	**0.1 ± 0.0**
